# Prognostic impact of pre-interventional culprit artery thrombolysis in myocardial infarction (TIMI) flow in patients with ST-segment elevation myocardial infarction treated by primary percutaneous coronary intervention

**DOI:** 10.1186/s43044-022-00289-3

**Published:** 2022-06-27

**Authors:** Raouf Shaaban, Adel El Etriby, Diaa Kamal, Ahmad E. Mostafa

**Affiliations:** Cardiology Department, Ain Shams University, 38 Ramsis Street, El Abbaseya, Cairo, Egypt

**Keywords:** STEMI, TIMI Flow, Primary PCI, Prognosis

## Abstract

**Background:**

Primary percutaneous coronary intervention (PCI) is considered the most preferred strategy in ST-segment elevation myocardial infarction (STEMI). However, the prognostic role of spontaneous re-canalization in STEMI patients is still not clear. The purpose of this study is to evaluate the impact of pre-procedural TIMI flow grade in the culprit coronary artery on the short and long term prognosis in Egyptian patients presented with STEMI and treated with primary PCI.

**Results:**

A dual center, prospective observational study that was conducted in the period from January 2019 till June 2020 and enrolled 150 STEMI patients presented within 24 h from onset of chest pain. Initial angiography was done with analysis of TIMI flow grade in the infarct related artery. Of the 150 enrolled patients; 93 patients (62%) were found to have initial TIMI flow grade 0 (group A) and 57 patients (38%) had initial TIMI flow grade I–III (group B). There was a strong association between cardiac mortality and pre-procedural TIMI flow grade. 12 mortalities (8% of total study population) were recorded during our study period; in-hospital mortality was reported in 7 patients in group A, yet no mortalities were recorded in-hospital in group B (*P* value = 0.033). At 1 year follow up; 5 mortalities were recorded in group A with no mortalities at all in group B (*P* value = 0.005). There was a trend towards an increase in acute heart failure incidence in group A yet no statistically significant value was achieved (*P* value = 0.112). Target lesion revascularization was reported in 8 patients in group A and in only 3 patients in group B (*P* value 0.446).

**Conclusions:**

Despite the evolution in primary PCI strategies and the continuous advancement in anti-thrombotic treatment; pre-interventional infarct related artery TIMI flow grade I–III is associated with better in hospital and 1 year outcome, specifically significantly lower cardiac mortality compared to patients who had TIMI flow grade 0 at initial angiography.

## Background

Primary percutaneous coronary intervention (PCI) has been shown to improve the outcome of patients with ST-segment elevation myocardial infarction (STEMI) in comparison to thrombolysis, mainly due to restoration of angiographic thrombolysis in myocardial infarction (TIMI) III flow grade in the vast majority of patients with further improvement of adjunctive pharmacological agents and mechanical devices. However, the prognostic role of early spontaneous re-canalization before undergoing angioplasty in these patients has not been thoroughly investigated. Although there is an excellent outcome conferred by primary PCI in patients with STEMI, the prognostic role of spontaneous re-canalization in these patients is still not clear [1].

Previous clinical studies have shown that the initial TIMI flow grade in the culprit coronary artery was an independent predictor of better one year survival in STEMI patients and that the TIMI flow grade 0 before primary PCI is associated with a worse clinical outcome. However, it is unclear whether the same is true with modern guideline-adherent therapy and reperfusion strategies [2–4].

The purpose of this study is to evaluate the impact of pre-procedural TIMI flow grade in the culprit coronary artery on the short and long term prognosis in Egyptian patients presented with STEMI and treated with primary PCI.

## Methods

Our study was a cohort, prospective and dual-center observational study. We started recruiting patients in January 2019 till June 2020. The study was conducted on 150 patients presenting with STEMI from both genders within 6 h from symptoms onset or between 6 and 24 h if they have continuous signs and symptoms of ongoing ischemia. Coronary angiography was done and coronary blood flow was assessed using TIMI flow grade. Patients with past ischemic history or patients who received thrombolytic therapy were excluded from the study.

Approval of university ethical committee was taken (MD 378/2018, FWA 000017585), and informed consent was obtained from all enrolled patients. All patients were subjected to the following: (a) detailed medical history with special emphasis on chest pain analysis, (b) general examination and local cardiac examination with assessment of Killip class, (c) 12 leads ECG within 10 min from first medical contact, (d) blood samples included (Complete Blood picture, Kidney function tests, liver function tests, Lipid profile, glycated Hemoglobin and cardiac enzymes including high sensitive troponin), (e) Trans-thoracic echocardiography: a rapid bed-side echo to assess left ventricular ejection fraction, resting wall motion abnormalities, and screen for mechanical complications, (f) pre-loading with 300 mg Aspirin orally and a P2Y12 inhibitor (either Clopidogrel 600 mg or Ticagrelor 180 mg orally according to availability). Coronary angiography was done via right radial or right femoral approaches according to the operator experience and tools availability with commenting on initial TIMI flow grade in the infarct related artery, non-culprit lesions, methods of antegrade flow restoration and glycoprotein IIb/IIIa inhibitor usage if done. Drug eluting stents were implanted in all indicated patients with sizing according to operator judgment. Final reporting on post PCI TIMI flow grade was mentioned.

After initial angiography; patients were divided into 2 groups: group (A) with initial TIMI flow grade 0 (107 patients ~ 62% of study population) and Group (B) with initial TIMI flow grade I-III (43 patients ~ 38% of study population). Our primary end point was occurrence of major adverse cardiac events (MACE) in the form of: non-fatal stroke, acute heart failure, target lesion revascularization and cardiac death either in-hospital or during the one year follow up period.

### Statistical analysis

Data were collected, revised, coded and entered to the Statistical Package for Social Science (IBM SPSS) version 23. The quantitative data were presented as mean, standard deviations and ranges when parametric and median, inter-quartile range (IQR) when data found non-parametric. Also, qualitative variables were presented as number and percentages. The comparison between groups with qualitative data was done by using *Chi-square test* and/or *Fisher exact test* when the expected count was found less than 5%.The comparison between two groups with quantitative data and parametric distribution were done by using *Independent t test.* While the comparison between two groups with quantitative data and non-parametric distribution was done by using *Mann–Whitney test.* The comparison between two paired groups with quantitative data and parametric distribution were done by using *Paired t test*. While the comparison between two paired groups with quantitative data and non-parametric distribution was done by using *Willcoxon test.* The confidence interval was set to 95% and the margin of error accepted was set to 5%. So, the *P* value was considered significant if *P* < 0.05.

## Results

Of the 150 studied patients, 126 patients (84%) were males and 24 patients (16%) were females with age ranged from 23 to 76 years with mean ± SD of 53.87 ± 9.64. Demographic and clinical data of our study population are shown in Table 1. After comparing group A and group B patients, baseline characteristics were generally similar apart from chest pain onset as shown in Table 2. Right femoral access was used in 78 patients (52%) and RT radial access was used in 71 patients (47%) and a single case was done via left radial access. LAD artery was found to be the most common infarct related artery with 71 patients (47.3%). Achieving final TIMI III flow grade was statistically significant when comparing both groups with *P* value = 0.016. Details of the procedural data are shown in Table 3. Laboratory and echocardiography data are shown in Table 4.

**Table 1:** Demographic and clinical data of the studied patients

	No. = 150
Age	53.87 ± 9.64
Male gender	126 (84%)
DM	76 (50.7%)
HTN	87 (58%)
Dyslipidemia	112 (74.7%)
Smoker	96 (64%)
FH of IHD	33 (22%)
*Killip class*	
I	100 (66.7%)
II	44 (29.3%)
III	6 (4%)
*Preloading antiplatelet*	
Ticagrelor	75 (50%)
Clopidogrel	75 (50%)
Chest pain onset (h)	3 (2–6)
*ECG*	
Inferior	60 (40%)
Anterior	74 (49.3%)
Posterior	12 (8%)
Lateral	4 (2.7%)
Systolic blood pressure	129.77 ± 16.73
Diastolic blood pressure	80.83 ± 10.97
Heart rate	84.33 ± 12.28

DM, diabetes mellitus; FH of IHD, family history of ischemic heart disease; HTN, hypertension

**Table 2:** Relation between pre TIMI flow grade and demographic/clinical data of the studied patients

	Pre TIMI		Test value	*P* value	Sig
	TIMI (0)	TIMI (I–III)			
	No. = 107	No. = 43			
Age	53.85 ± 9.43	53.91 ± 10.06	− 0.039•	0.969	NS
Male Gender	80 (86.0%)	46 (80.7%)	0.744*	0.388	NS
DM	51 (54.8%)	25 (43.9%)	1.704*	0.192	NS
HTN	52 (55.9%)	35 (61.4%)	0.437*	0.508	NS
Dyslipidemia	71 (76.3%)	41 (71.9%)	0.364*	0.546	NS
Smoking	61 (65.6%)	35 (61.4%)	0.269*	0.604	NS
FH of IHD	18 (19.4%)	15 (26.3%)	0.998*	0.318	NS
*Killip class*					
I	60 (64.5%)	40 (70.2%)	1.379*	0.502	NS
II	28 (30.1%)	16 (28.1%)			
III	5 (5.4%)	1 (1.8%)			
*Preloading*					
Ticagrelor	44 (47.3%)	31 (54.4%)	0.707*	0.400	NS
Clopidogrel	49 (52.7%)	26 (45.6%)			
CP Onset (h)	4 (3–6)	3 (2–4)	− 2.361^≠^	0.018	S
*ECG*					
Inferior	35 (37.6%)	25 (43.9%)	0.571	0.449	NS
Anterior	46 (49.5%)	28 (49.1%)	0.002	0.964	NS
Posterior	9 (9.7%)	3 (5.3%)	0.936	0.333	NS
Lateral	3 (3.2%)	1 (1.8%)	0.295	0.587	NS
Systolic blood pressure	130.16 ± 18.00	129.12 ± 14.55	0.368•	0.713	NS
Diastolic blood pressure	81. 24 ± 11.74	80.18 ± 9.63	0.574•	0.567	NS
Heart rate	83.97 ± 11.78	84.91 ± 13.14	− 0.456•	0.649	NS

*P* value > 0.05: Non significant (NS); *P* value < 0.05: Significant (S); *P* value < 0.01: Highly significant (HS)

Data is presented as mean ± SD and percentage

DM, diabetes mellitus; FH of IHD, family history of ischemic heart disease; HTN, hypertension

^*^Chi-square test; ^•^Independent *t* test; ^≠^Mann–Whitney test

**Table 3:** Relation between pre TIMI flow grade with procedural data of the studied patients

	Pre TIMI		Test value	*P* value	Sig
	TIMI (0)	TIMI (I–III)			
	No. = 107	No. = 43			
*Access*					
RT radial	40 (43.0%)	31 (54.4%)	3.769*	0.152	NS
RT femoral	53 (57.0%)	25 (43.9%)			
LT radial	0 (0.0%)	1 (1.8%)			
DTB time (min)	37.01 ± 10.87	38.77 ± 9.84	− 0.998•	0.320	NS
*IRA*					
Negative	0 (0.0%)	4 (7.0%)	6.705	0.009	HS
RCA	31 (33.3%)	21 (36.8%)	0.192	0.661	NS
LAD	45 (48.4%)	26 (45.6%)	0.109	0.741	NS
LCX	11 (11.8%)	6 (10.5%)	0.060	0.806	NS
OM	3 (3.2%)	0 (0.0%)	1.876	0.170	NS
D1	2 (2.2%)	0 (0.0%)	1.242	0.265	NS
SVG to PDA	1 (1.1%)	0 (0.0%)	0.617	0.432	NS
Non culprit lesions	45 (50.6%)	19 (35.2%)	3.214*	0.073	NS
*Antegrade flow restoration*					
Negative	0 (0%)	11 (19.3%)	26.188*	0.000	HS
Wiring	4 (4.3%)	8 (14.0%)			
PTCA	73 (78.5%)	25 (43.9%)			
Aspiration	15 (16.1%)	13 (22.8%)			
Post dilatation	3.36 ± 0.41	3.39 ± 0.48	− 0.215•	0.831	NS
GP IIb/IIIa inh	28 (30.1%)	13 (22.8%)	0.948*	0.330	NS
*Post TIMI*					
I	6 (6.5%)	0 (0.0%)	8.231*	0.016	S
II	17 (18.3%)	4 (7.0%)			
III	70 (75.3%)	53 (93.0%)			
*Blush grade*					
I	7 (7.5%)	0 (0.0%)	8.393*	0.015	S
II	16 (17.2%)	4 (7.0%)			
III	70 (75.3%)	53 (93.0%)			

*P* value > 0.05: Non significant (NS); *P* value < 0.05: Significant (S); *P* value < 0.01: Highly significant (HS)

Data is presented as mean ± SD and percentage

DTB, door to balloon; IRA, infarct related artery; GP IIb/IIIa inh., glycoprotein IIb/IIIa inhibitors; PTCA, percutaneous transluminal coronary angioplasty; RT, right; LAD, left anterior descending; LCX, left circumflex; RCA, right coronary artery; D1, first diagonal branch; SVG to PDA, saphenous venous graft to posterior descending artery

^*^Chi-square test; ^•^Independent *t* test

**Table 4:** Relation between Pre TIMI flow grade and laboratory results / echocardiographic date of the studied patients

	Pre TIMI		Test value	*P* value	Sig
	TIMI (0)	TIMI (I–III)			
	No. = 107	No. = 43			
*CBC*					
Hemoglobin	14.02 ± 1.68	13.96 ± 1.58	0.214•	0.831	NS
TLC	10.93 ± 3.38	9.87 ± 2.71	1.999•	0.047	S
Platelets	256.25 ± 66.09	265.89 ± 58.71	− 0.905•	0.367	NS
*Kidney function tests*					
Creatinine	1.09 ± 0.22	1.05 ± 0.27	0.834•	0.406	NS
Urea	38.38 ± 10.57	37.75 ± 10.16	0.355•	0.723	NS
*Cardiac enzymes/troponin*					
CK	2112 (1283–2700)	1832 (1402–2636)	− 0.501^≠^	0.616	NS
CKMB	220 (155–278)	198 (139–266)	− 1.225^≠^	0.220	NS
Troponin	1874 (1455–2844)	1950 (1267–2831)	− 0.426^≠^	0.670	NS
INR	1.06 ± 0.08	1.03 ± 0.08	2.017•	0.046	S
*Liver function tests*					
ALT	45 (35–60)	40 (34–48)	− 1.964^≠^	0.049	S
AST	120 (78–176)	88 (54–122)	− 2.740^≠^	0.006	HS
*Lipid profile*					
Cholesterol	222.87 ± 49.09	220.56 ± 41.11	0.297•	0.767	NS
LDL	157 (113–180)	152 (95–184)	− 0.174^≠^	0.862	NS
Triglycerides	145 (124–197)	155 (132–210)	− 0.951^≠^	0.342	NS
Initial EF (%)	43.87 ± 8.57	47.05 ± 7.55	− 2.305•	0.023	S
Hospital stay (days)	3.66 ± 0.97	3.40 ± 1.02	1.522•	0.130	NS

*P* value > 0.05: Non significant (NS); *P* value < 0.05: Significant (S); *P* value < 0.01: Highly significant (HS)

^•^Independent *t* test; ^≠^Mann–Whitney test; *Chi-square test

Data is presented as mean ± SD and median IQR

EF, ejection fraction; HF, heart failure; MACE, major adverse cardiac events; TLC, total leukocetic count; TLR, target lesion revascularization; INR, International normalized ratio; AST, aspartate aminotransferase; ALT, alanine transaminase; LDL, low density lipoprotein

As shown in Table 5, our study reported that out of the 150 enrolled patients; there were 121 (80%) patients that had no MACE. On the other hand; 29 (20%) patients developed adverse events in the form of: acute heart failure (10 patients ~ 6.6%), non-fatal stroke (1 patient ~ 0.66%) which occurred during the follow up period and cardiac death (12 patients ~ 12.6%) either in hospital or during the one year follow up period.

**Table 5:** Follow up results of the studied patients

	Pre TIMI		Test value	*P* value	Sig
	TIMI (0)	TIMI (I–III)			
	No. = 93	No. = 57			
*Initial EF (%)*					
Mean ± SD	43.87 ± 8.57	47.05 ± 7.55	− 2.305^•^	0.023	S
Range	20–75	30–63			
*Hospital stay (days)*					
Mean ± SD	3.66 ± 0.97	3.40 ± 1.02	1.522^•^	0.130	NS
Range	2–6	2–8			
*In-hospital MACE*					
Negative	80 (86.0%)	54 (94.7%)	2.817*	0.093	NS
Mortality	7 (7.5%)	0 (0.0%)	4.500*	0.033	S
TLR	0 (0.0%)	0 (0.0%)	NA	NA	NA
HF admission	6 (6.5%)	2 (3.5%)	0.606*	0.436	NS
CABG	0 (0.0%)	1 (1.8%)	1.643*	0.199	NS
TIA	0 (0.0%)	0 (0.0%)	NA	NA	NA
*Follow up EF (%)*					
Mean ± SD	45.42 ± 9.27	50.12 ± 7.69	− 3.193^•^	0.002	HS
Range	22–70	30–65			
*MACE FUP*					
Negative	67 (72.0%)	54 (94.7%)	11.670*	0.000	HS
Mortality	12 (12.9%)	0 (0.0%)	7.994*	0.005	HS
TLR	8 (8.6%)	3 (5.3%)	0.580*	0.446	NS
HF admission	4 (4.3%)	0 (0.0%)	2.519*	0.112	NS
CABG	1 (1.1%)	0 (0.0%)	0.617*	0.432	NS
TIA	1 (1.1%)	0 (0.0%)	0.617*	0.432	NS

*P* value > 0.05: Non significant (NS); *P* value < 0.05: Significant (S); *P* value < 0.01: Highly significant (HS)

Data is presented as mean ± SD and percentage

EF, ejection fraction; HF, heart failure; MACE, major adverse cardiac events; TIA, transient ischemic attack, TLR, target lesion revascularization

^*^Chi-square test; ^•^Independent *t* test

There was a strong association between cardiovascular mortality and pre-interventional TIMI flow grade. In-hospital mortality was reported in 7 patients in group A, yet no mortalities were recorded in-hospital in group B yielding a statistically significant *P* value of 0.033.

After one year of follow up; mortality was much higher in group A (12 mortalities) than in group B which recorded no mortalities at all in the follow up period with statistically significant *P* value 0.005. There was a trend towards an increase in acute heart failure incidence in group A yet no statistically significant value was achieved. Target lesion revascularization was reported in 8 patients in group A and in only 3 patients in group B (*P* value 0.446).

The univariate and multivariate analysis are shown in Table 6. The multivariate analysis adjusting for differences in demographic data, clinical data, procedural data, laboratory results, pre-interventional TIMI flow grade and hospital stay yielded similar results favoring better outcomes with pre-interventional TIMI flow grade I-III.

**Table 6:** Univariate and multivariate by backward Wald logistic regression analysis for factors associated with positive MACE at follow up

	Univariate				Multivariate			
	*P* value	Odds ratio (OR)	95% C.I. for OR		*P* value	Odds ratio (OR)	95% C.I. for OR	
			Lower	Upper			Lower	Upper
Age > 49	0.010	5.132	1.469	17.923	0.036	5.743	1.124	29.355
Systolic < = 110	0.000	6.125	2.494	15.041	–	–	–	–
Diastolic < = 85	0.032	2.889	1.097	7.605	–	–	–	–
Killip class > = II	0.000	5.516	2.316	13.136	–	–	–	–
PTCA	0.013	4.110	1.346	12.552	–	–	–	–
GP IIb/IIIa	0.006	3.249	1.396	7.563	–	–	–	–
Pre TIMI < I	0.002	6.985	2.006	24.324	0.010	6.337	1.566	25.642
Creat > 1	0.007	3.368	1.383	8.201	–	–	–	–
Urea > 54	0.000	13.162	3.698	46.855	–	–	–	–
Trop > 2490	0.001	4.187	1.793	9.781	0.026	3.326	1.151	9.608
ALT > 59	0.001	4.649	1.916	11.285	–	–	–	–
AST > 132	0.002	3.778	1.630	8.754	–	–	–	–
EF < = 47%	0.004	5.169	1.691	15.799	–	–	–	–
Hospital stay > 4 days	0.000	7.929	2.808	22.385	0.016	4.398	1.324	14.616

*P* value > 0.05: Non significant (NS); *P* value < 0.05: Significant (S); *P* value < 0.01: Highly significant (HS)

ALT, alanine transaminase; AST, aspartate aminotransferase; C.I., confidence interval; EF, ejection fraction; GP IIb/IIIa inh., Glycoprotein IIb/IIIa inhibitors; OR, odds ratio; PTCA, percutaneous transluminal coronary angioplasty

^*^Chi-square test; ^•^Independent *t* test

## Discussion

In the present study; pre-interventional TIMI flow grade was found to be a strong independent factor for survival in STEMI patients treated by primary PCI. The importance of early spontaneous recanalization is justified by the larger extension of myocardial loss observed with poor pre-interventional TIMI flow grade. Evidence of patent infarct related artery both prior to and after primary PCI is supposedly to have better clinical outcomes. Compared with early spontaneous recanalization, delayed recanalization is associated with an older, organized intra-coronary thrombus which will result in more impairment in myocardial perfusion limiting myocardial salvage [5].

The TIMI flow grade was initially established to ensure a uniform method of documenting epicardial perfusion in coronary angiography. We investigated the prognostic role of early re-canalization as assessed by TIMI flow grade in the pre-interventional coronary angiography in these patients.

The diagnosis of STEMI is the clinical representation of an acute occlusion of an epicardial coronary artery and thus it’s not surprising that the majority of patients with STEMI have a TIMI flow grade 0 at time of initial angiography. In some patients with STEMI, spontaneous recanalization occurs before angiography and PCI. Stone et al. analyzed the four Primary Angioplasty in Myocardial Infarction (PAMI) trials with 2507 enrolled patients and showed that normal pre-interventional TIMI flow grade is an independent determinant of survival [3].

This finding was confirmed by De Luca et al., in whose study initial TIMI flow grade was significantly correlated with one-year mortality in high-risk TIMI risk score patients [4].

These results were further supported by the databases from the CADILLAC and HORIZONS-AMI trials in which an initial TIMI I-III flow grade was independently associated with a 65% decrease in 1-year all-cause mortality. They also showed that initial TIMI flow grade I–III was associated with a significant improvement of final TIMI flow grade which in terms shows a better perfusion at the end of PCI and was correlated with better outcomes and lower 1 year mortality [2]. Our data has also proved the same finding of having initial TIMI flow grade I–III will mostly result in final TIMI flow grade III which has better follow up results. In contrary to patients having initial TIMI flow grade 0 that showed lesser chances of ending up with TIMI III flow grade after PCI and final poorer net clinical outcome.

Total occlusion of infarct related artery was noted in almost 70% of patients in most of clinical trials investigating the clinical impact of pre-interventional TIMI flow grade [2–4]. In our studied population; we recorded 62% of patients with pre-interventional TIMI flow grade 0 (Group A) and 38% with TIMI flow grade I–III (Group B), with initial TIMI III flow grade being the least recorded at about 4% of studied population.

We found that group A patients are still having poorer early clinical outcomes compared to those with initial TIMI flow grade I-III and this was still valid till the later period in the one year follow up. 12 mortalities (8% of total study population) were recorded during our study period; 7 of them (4.7%) were recorded in-hospital and 5 mortalities (3.3%) were recorded over the one year follow up. In-hospital mortalities occurred in those patients who had Killip class II–III at time of presentation with longer chest pain onset compared to the other studied patients.

In comparison to mortalities recorded in a study done by Rakowzki et al., our study showed higher percentage of cardiac mortalities either in-hospital or during the follow up period. This may be attributed to longer onset of chest pain as mentioned in our data. However, both studies were similar in that the mortality was higher in patients with TIMI 0 flow grade than that in patients with TIMI I-III flow grade [6]. This was also consistent with the data presented by Pravda et al. in the ACSIS registry [7].

Our data was also consistent with what Stone et al. concluded regarding the pre-interventional TIMI flow grade 0 and both in-hospital and one year follow up mortality rates [3].

On contrary, Bauer et al. proved no significant correlation between pre-interventional TIMI flow grade and adverse cardiac events in the ATLANTIC study which enrolled 1862 STEMI patients. However; their study design enrolled only low risk patients and excluded any patient presented with cardiogenic shock or severe hemodynamic instability although the benefit of early recanalization is more pronounced in higher risk patients [8].

Brener et al. showed paradoxical results in contrary to all clinical trials investigating the pre-interventional TIMI flow grade impact in STEMI patients. They mentioned that patients with TIMI III flow at initial angiography had higher clinical risk features than that with TIMI flow grade 0 with diabetes mellitus, smoking and longer period to primary PCI as independent predictors to pre-interventional TIMI III flow [2].

In our study, we didn’t have any clinical or demographic factor with that could significantly affect pre-interventional TIMI flow grade except for chest pain onset which has some contributing factors especially in Egypt that can lengthen the time to first medical contact and proper management strategy including the primary PCI decision. This delay is concerning and included both patients’ related factors and system related factors. This elaborates the importance of public health awareness of recognizing alarming symptoms of acute myocardial infarction as well as importance of immediate medical care.

Based on the importance of time delay to primary PCI and the beneficial effect of early recanalization, the concept of pharmacologically induced recanalization was established. Theoretical advantages of early infarct related artery patency included easier guidewire crossing, better stent sizing selection and lower thrombus burden with lower risk of distal embolization.

This strategy has been tested in many clinical trials using thrombolytics or GP IIb/IIIa inhibitors or combination of both, but hasn’t shown clinical benefit over primary PCI mainly because of increased bleeding events [9].

Our data warrants intensified risk factors control with pharmacological and non-pharmacological management together with stricter follow up and surveillance in STEMI patients who had initial TIMI grade 0 as they showed to have a higher incidence of adverse effects and more importantly higher mortality rates.

## Conclusions

Despite the evolution in primary PCI strategies and the continuous advancement in anti-thrombotic treatment; pre-interventional infarct related artery TIMI flow grade I-III is still associated with better clinical outcomes at 1 year follow up, specifically lower cardiac mortality compared to patients who had TIMI flow grade 0 at initial angiography.

### Limitations

The present study has number of limitations. Our study recruited 150 patients; we recommend increasing the number in future studies. We followed up our patients for one year; a longer follow up period maybe needed to insure the clinical outcomes.

Patients with past ischemic history were excluded from our study so our results couldn’t be applied to this category of patients.

Detection of myocardial infarction size and distribution could have been done using cardiac magnetic resonance imaging.

## Data Availability

The datasets used and analyzed during the current study are available from the corresponding author on reasonable request.

## Abbreviations

ALT: Alanine transaminase
AST: Aspartate aminotransferase
CK: Creatine kinase
CK-MB: Creatinine kinase myocardial band
D1: First diagonal branch
DM: Diabetes mellitus
DTB: Door to balloon
ECG: Electrocardiogram
EF: Ejection fraction
FH of IHD: Family history of ischemic heart disease
GP IIb/IIIa inh.: Glycoprotein IIb/IIIa inhibitors
HF: Heart failure
HTN: Hypertension
INR: International normalized ratio
IRA: Infarct related artery
LAD: Left anterior descending
LCX: Left circumflex
LDL: Low density lipoprotein
MACE: Major adverse cardiac events
PCI: Percuteanous coronary intervention
PTCA: Percutaneous transluminal coronary angioplasty
RCA: Right coronary artery
RT: Right
STEMI: ST-elevation myocardial infarction
SVG to PDA: Saphenous venous graft to posterior descending artery
TIA: Transient ischemic attack
TIMI: Thrombolysis in myocardial infarction
TLC: Total leukocyte count
TLR: Target lesion revascularization
